# A New Asynchronous Parallel Algorithm for Inferring Large-Scale Gene Regulatory Networks

**DOI:** 10.1371/journal.pone.0119294

**Published:** 2015-03-25

**Authors:** Xiangyun Xiao, Wei Zhang, Xiufen Zou

**Affiliations:** 1 School of Mathematics and Statistics, Wuhan University, Wuhan, China; 2 School of Science, East China Jiaotong University, Nanchang, China; Huazhong University of Science and Technology, CHINA

## Abstract

The reconstruction of gene regulatory networks (GRNs) from high-throughput experimental data has been considered one of the most important issues in systems biology research. With the development of high-throughput technology and the complexity of biological problems, we need to reconstruct GRNs that contain thousands of genes. However, when many existing algorithms are used to handle these large-scale problems, they will encounter two important issues: low accuracy and high computational cost. To overcome these difficulties, the main goal of this study is to design an effective parallel algorithm to infer large-scale GRNs based on high-performance parallel computing environments. In this study, we proposed a novel asynchronous parallel framework to improve the accuracy and lower the time complexity of large-scale GRN inference by combining splitting technology and ordinary differential equation (ODE)-based optimization. The presented algorithm uses the sparsity and modularity of GRNs to split whole large-scale GRNs into many small-scale modular subnetworks. Through the ODE-based optimization of all subnetworks in parallel and their asynchronous communications, we can easily obtain the parameters of the whole network. To test the performance of the proposed approach, we used well-known benchmark datasets from Dialogue for Reverse Engineering Assessments and Methods challenge (DREAM), experimentally determined GRN of Escherichia coli and one published dataset that contains more than 10 thousand genes to compare the proposed approach with several popular algorithms on the same high-performance computing environments in terms of both accuracy and time complexity. The numerical results demonstrate that our parallel algorithm exhibits obvious superiority in inferring large-scale GRNs.

## Introduction

The reconstruction of gene regulatory networks (GRNs) from high-throughput genome-wide data can help improve our understanding of molecular regulation events and is one of the most important issues in systems biology, which explicitly characterizes regulatory processes in the cell [1, 2]. Because of the advances in high-throughput technologies, the size of the GRNs we need to understand is becoming incredibly larger than before and extremely complicated. Recently, the Dialogue for Reverse Engineering Assessments and Methods (DREAM) project provided a platform for researchers to develop new efficient computation algorithms to infer GRNs [3]. For the reverse engineering of GRNs, various state-of-the-art approaches have been developed to improve the accuracy and scalability of network inference review in [4], including correlation based estimation methods [5], model-based methods [6, 7, 8], and mutual information(MI)-based methods, such as CMI [9], MI-CMI [10], MI3 [11] and PCA-CMI [12] and IPCA-CMI [13]. Furthermore, NARROMI [14] combined regression-based optimization and information theory- based MI to achieve improved accuracy.

However, the above approaches suffer from two limitations when handling networks with large numbers of genes. One limitation is that the computation cost increases exponentially with the number of genes. The second is that the accuracy of these approaches in large-scale problems with high sparsity is not satisfactory. In recent years, there have been efforts to address these difficulties. Qin at al. applied LASSO-type regularization methods to enhance the prediction accuracy [15]. Lee at al. developed a parallelizing hybrid GA-PSO optimization method to lower the time complexity [16], but in their numerical experiments, all three datasets contain no more than 125 genes. Because the sparsity level of the large-scale GRNs is much higher than for small-scale GRNs, false positives also increase remarkably when a similar correlation cutoff is used to predict the GRNs. In this study, we used the two features of large-scale GRNs, i.e., their sparsity and modularity, to design a new parallel framework to infer GRNs. We first decomposed whole large-scale GRNs into many small modular subnetworks and used module-based optimization and asynchronous communications to identify the parameters of the GRNs. Finally, we used famous benchmark datasets from DREAM with thousands of genes to compare with several popular algorithms on the same high-performance computing environments. The results show that the proposed approach can be successfully used to infer large-scale GRNs with high accuracy, and the computation time can be greatly reduced.

## Methods

**Fig 1:**
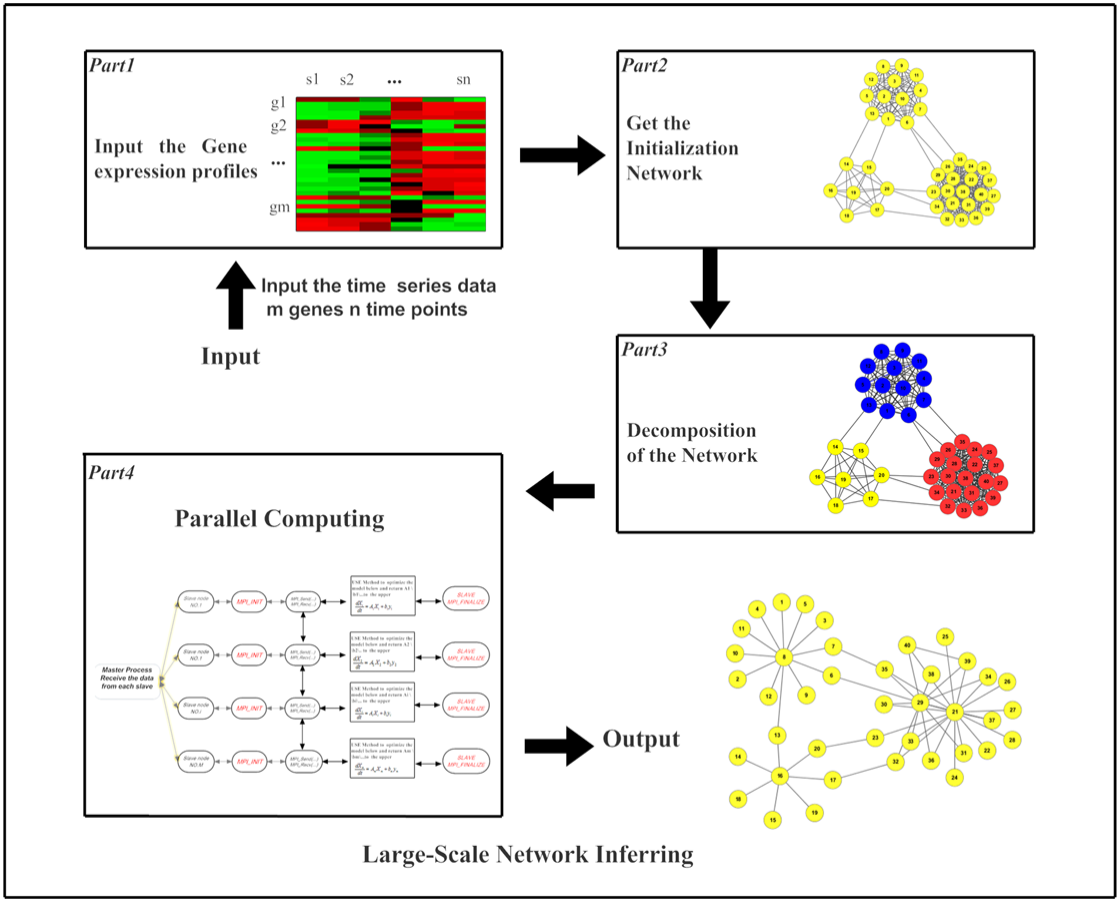
Outline of reconstructing large-scale gene networks by using the LSGPA.

The proposed approach, namely the LSGPA, includes four parts (Fig. 1). The first part is to interpolate and normalize the gene expression data. The second is to build the initialization network based on the mutual information (MI) presented in section 2.1. In the third part, we have proposed a module-based decomposition to split the whole network into a large number of subnetworks, as detailed in section 2.2. Finally, the fourth part includes the parallel design and algorithm, which is presented in sections 2.3 and 2.4.

### 2.1 Mutual information

The IFN concentration was measured by a bioassay based on the IFNα/β-mediated Mutual information (MI) is generally used as a powerful criterion for measuring the relationship between two genes *X* and *Y* in biological networks inference. MI has been widely used to construct GRNs from gene expression data [17].

For two discrete variables (genes) *X* and *Y*, the joint entropy *H(X*,*Y*) of *X* and *Y* can be denoted by

$$H(X,Y)=-\sum_{x\in X,y\in Y}p(x,y)logp(x,y)$$ (1)

where *p(x*,*y*) is the joint probability of *x* in *X* and *y* in *Y*. In light of the equation displayed above, MI can be denoted as follows

I(X,Y)=H(X)+H(Y)-H(X,Y) (2)

In computational simulation, we can just use the following equivalent formula [12].

$$I(X,Y)=0.5\cdot log\frac{\left|C(X)|\cdot |C(Y)\right|}{\left|C(X,Y)\right|}$$ (3)

where *C* is the covariance matrix, and |*C*(*X*)| is the determinant of matrix *C*.

If *X* and *Y* are independent, the value will be relatively low, while a high MI value indicates that there may be a close relationship between the genes.

### 2.2 Module-based decomposition

In recent years, many excellent algorithms have been proposed to detect the modules of complex networks [18–23]. Most of these algorithms were only used to identify small and highly intra-connected clusters in a network, without clustering all the nodes. However, in the real biological world, GRNs often contain thousands of nodes and perform in a sparse way [24]. More recently, Glay proposed an algorithm to display and analyze large and sparse biological networks [25].

In this study, we proposed an improved approach to detect and split the modules. There are three steps.

**Step1**. Building the initialization networks and obtaining the network adjacency matrix

First, we construct initial networks by calculating the MI according to formula (3). By calculating the MI values between all pairs of variables, we obtain a weighted matrix *A = (a*_*ij*_), in which every element represents a relationship between two genes.

Second, we select a threshold “*λ*” to cut off edges with low value. In other words, if element *a*_*ij*_ is greater than *λ*, then we set its value to 1 and let *a*_*ji*_ = *a*_*ij*_ = 1. Otherwise, no edges will be considered, i.e., *a*_*ji*_ = *a*_*ij*_ = 0. We performed simulation experiments to mine for information about how to select *λ*, and the detailed comparisons are displayed in S1 Table and S2 Fig. In this study, we set the MI threshold *λ* to 1.2.

Finally, a new network adjacency matrix can be used in clustering.

**Step 2**. Clustering and modularizing the large GRNs.

First, the network adjacency matrix is transformed into an interaction matrix with two columns. The first column contains source nodes and the second column contains target nodes. Second, we use Glay’s algorithm to cluster large GRNs and obtain *M* modules named *C*_*i*_ (*i* = 1, 2,…, *M*).

**Step 3**. Using the optimization algorithm to ensure that each node in the network belongs to one of all modules.

As we all know, many algorithms have been engineered to identify small and highly intra-connected clusters in a network, without clustering all the nodes [3, 23]. In Step 1, when selecting a threshold to remove edges, certain vectors (genes) might have been "kicked out" from the whole network. To ensure the integrity of the networks to be inferred, we provide the following optimization method.

Because all the genes that were removed must be returned to the whole network, we compute the mutual information value between each "kicked out" node and the nodes in all modules. For example, if gene *x* was removed from the whole network, the MI value should be calculated between *x* and each node in all modules *C*_*i*_ (*i* = *1*, *2*,…, *M*). Then, we use formula (4) to search for the index with the maximum MI value between *x* and *C*_*i*_.

$$i_{0}=Index\left\{\underset{1\leq i\leq M}{\max}\left\{\underset{1\leq j\leq |C_{i}|}{\max}\left\{\underset{y_{ij}\in C_{i}}{MI}(x,y_{ij})\right\}\right\}\right\}$$ (4)

Therefore, gene *x* will be inserted into module $C_{i_{0}}$. Finally, all genes are split into modules without any isolated genes.

### 2.3 Mathematical Model and Optimization

Generally, a GRN consisting of N genes can be modeled by a set of ordinary differential equations (ODEs) [4, 15]. In this study, the ODE model in each cluster *C*_*i*_ can be described as follows.

$$\frac{dx_{i}^{j}(t)}{dt}=\sum_{k=1}^{T_{i}}a_{ik}^{j}x_{i}^{k}(t)+\sum_{l=1}^{H_{i}}b_{il}^{j}y_{i}^{l}(t)$$ (5)

The first term in the right of Equation. (5) represents the internal links between the same module *C*_*i*_ and the second term represents the external links from other modules *C*_*l*_(*l≠i*)to the module *C*_*i*_. *x* ^*j*^_*i*_(*t*) represents the express level of gene *j* at time *t* in module *C*_*i*_, *a* ^*j*^_*ik*_ represents the weight value of influence from gene *k* to gene *j* in module *C*_*i*_ and *T*_*i*_ is the gene number in the module *C*_*i*_. We denoted the set that links with module *C*_*i*_ as the Candidate_set ${\overset{-}{C}}_{i}$ of *C*_*i*_. The value *b* ^*j*^_*il*_ describes the weight value of gene *l* in the set ${\overset{-}{C}}_{i}$ to gene *j*, *y* ^*l*^_*i*_(*t*) is the expression level of gene *l* at time *t* in set ${\overset{-}{C}}_{i}$ and *H*_*i*_are the gene number in the set ${\overset{-}{C}}_{i}$. Therefore, the purpose of reconstructing the GRNs is to optimize all weight values *a* ^*j*^_*ik*_and *b* ^*j*^_*il*_in Equation. (5).

First, we take the difference for the left item of the above ODE equation.

$$\frac{dx_{i}^{j}(t)}{dt}\approx x_{i}^{j}(t)-x_{i}^{j}(t-1)=d_{i}^{j}(t)$$ (6)

Thus, we can obtain a linear system.

$$d_{i}^{j}(t)=\sum_{k=1}^{T_{i}}a_{ik}^{j}x_{k}^{j}(t)+\sum_{l=1}^{H_{i}}b_{il}^{j}y_{l}^{j}(t)$$ (7)

For the different time points tm, (*m* = 1, 2,…, *T*), we obtain the matrix form.

$$H_{i}P_{i}^{j}=D_{i}^{j}(t)$$ (8)

Where $X_{i}={\left(x_{k}^{i}(t_{m})\right)}_{T\times T_{i}},Y_{i}={\left(y_{l}^{i}(t_{m})\right)}_{T\times H_{i}},H_{i}=({\begin{array}{cc} X_{i} & Y \end{array}}_{i}),$ $P_{i}^{j}={\left(a_{i1}^{j},a_{i2}^{j},\cdots a_{iT_{i}}^{j},b_{i1}^{j},b_{i2}^{j},\cdots ,b_{iH_{i}}^{j}\right)}^{T},\;D_{{}_{i}}^{i}={\left(d_{i}^{j}(t_{1}),d_{i}^{j}(t_{2}),\cdots ,d_{i}^{j}(t_{T})\right)}^{T}.$

The identification of all unknown coefficients *P* ^*j*^_*i*_ for module *C*_*i*_ (*i* = 1, 2,…, *M*) and gene *j* in Equation. (8) can be transformed into the following least-square problem.

$$minQ=\underset{P_{i}^{j}}{min}||H_{i}P_{i}^{j}-D_{i}^{j}(t)|{|}_{2}^{2}$$ (9)

First, we calculate the condition number of matrix *H*_*i*_ in equation. (9). If it is relatively large, we can use the QR factorization to solve the (9) to obtain all coefficients *P* ^*j*^_*i*_. If the condition number of matrix *H*_*i*_ is small, the regularization method is used. Therefore, the following normal equations are obtained:

$$H_{i}^{T}H_{i}P_{i}^{j}=H_{i}^{T}D_{i}^{j}(t)\;$$ (10)

We use the Gauss elimination with pivoting to solve the linear systems for all modules in parallel.

### 2.4 Parallel Computing Algorithm

#### 2.4.1 Parallel computational environment

The Message Passing Interface (MPI) is a standardized and portable message-passing system designed by a group of researchers from academia and industry to function on a wide variety of parallel computers. The MPI provides a method based on a message passing parallel programming environment to communicate between the service processes. Its installation is presented in S1 Text. Generally, MPI has two main modes: Peer-to-peer mode and Master/Slaves mode. In this study, we choose Master/Slaves mode, and each slave can process one or several modules.

#### 2.4.2 Calculate the connections between modules

To achieve communication between different slaves and improve the accuracy of constructing GRNs, we need to select the edges between different modules more accurately. First, we calculate and rank all MI values of two different modules *C*_*n*_ and *C*_*m*_, respectively. Because of the sparsity of large-scale biological networks, we then select those the top *5%~ 10%* rankings of MI values as representatives of the connections between every two modules. In this way, all separated modules can be linked through those edges.

#### 2.4.3 Parallel design and asynchronous communications

**Fig 2:**
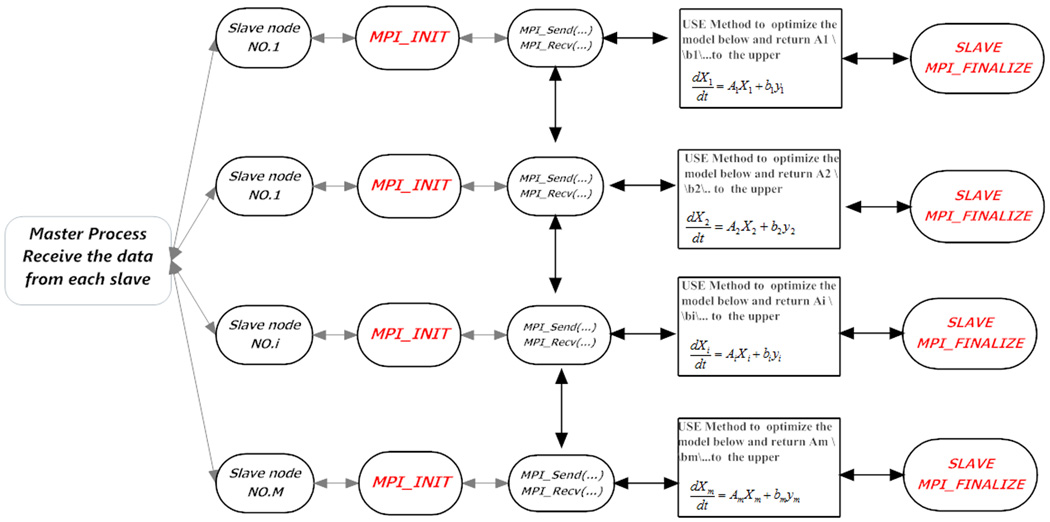
Outline of Parallel Computing.

The proposed parallel framework is depicted in Fig. 2.

**Step 1**. After finishing the splitting of network in Master, M subnetworks were sent to all Slaves.

For cases when the number of subnetworks/modules is larger than the number of computing nodes for the cluster system, we designed a special process to overcome the limitation of the number of SLAVES, which is described in S3 Text.

**Step 2**. For each module/Slave, we combine the ODE-based model and MLR to obtain the subnetworks and the connections among subnetworks. The algorithm is listed in Table 1.

**Table 1:** Algorithm 1.

**The pseudocode procedure for the parallel communications of the LSGPA**.
***for*** *Slave i* = 1 to *i* = *M* ***in Parrallel***
**→**.***Let*** *Slave*(i) ***MPI_Rev*** *dataset*(*i*) *from Master*, *C*_*i*_;
→.***MPI_Brocast*** *C*_*i*_ *to all Slaves for 1 to M*;
→.***Compute*** *I*(*x*,*y*) for every pair of genes (*x,y*);
→.*Give the* ***threshold*** *of MI values λ*, *if I*(*x*,*y*)<*λ then I(x*,*y) = 0*,
*else I(x*,*y) = 1 and edge (x*,*y) will be selected into C*_*i*_ (*Candidate_set*(*i*));
→.***MPI_Rev*** *C*_*i*_ *from Slaves1 to M*
***Compute*** *MI values for every pair of edges* (*x*,*y*_*j*_) *x*∈*C*_*i*_, *y*_*j*_∈*C*_*k*_,*k* = *1 to M*, *k*≠*i*;
***Rank*** *all the MI values*, *selected top* 5% *value for every pair of edges into Candidate_set of C*_*i*_.
→.*Give the paameter threshold θ*(**)
***for*** *j = 1 to T*_*i*_
Solving the normal equations $H_{i}^{T}\;H_{i}\;P_{i}^{j}\;=\;H_{i}^{T}\;D_{i}^{j}(t)$ or using the QR factorization;
to obtain parameters $P_{i}^{j}={\left(a_{i1}^{j},a_{i2}^{j},L,a_{iT_{i}}^{j},b_{i1}^{j},b_{i2}^{j},L,b_{iH_{i}}^{j}\right)}^{T}={\left(\beta_{1},\beta_{2},L,\beta 1,\beta_{T_{i}+H_{i}}\right)}^{T};$
***for*** *l = 1 to T*_*i*_ *+H*_*i*_
***if*** *β*_*l*_ *> θ*
*β*_*l*_ *= 1 and edge(y*_*k*_,*x*_*l*_) = 1;
***else***
*β*_*l*_ = 1 *and edge(y*_*k*_,*x*_*l*_) = 0;
***Endfor***
*Net(i).Matrix* $(K,:)={\left(\beta_{1},\beta_{2},L,\beta_{T_{i}+H_{i}}\right)}^{T};$
***Endfor***
→**MPI_Send** Net(i).Matrix to Master;
***Endfor***

**Step 3**. We send all network topologies represented by matrixes from modules/Slaves to the Master and recombine them as a whole network.

## Results

All the numerical experiments were conducted on a high-performance parallel computing environment with a unified cluster system, which includes the Dawning cluster and HP cluster. The Dawning cluster has a peak speed of 19.64 TFlops and includes 93 computing nodes and two management nodes. Each node includes 2 CPUs, and each CPU includes 2 cores. The clock speed is 2.2 GHz, and the memory of each node is 128 GB. The HP cluster has a peak speed of 2.675 Tflops and includes 76 computing nodes and two management nodes. Each node includes 2 CPUs, and each CPU includes 2 cores. The clock speed is 2.2 GHz, and the memory of each node is 4 GB.

### 3.1 Datasets used in this study

As a well-known dataset, the DREAM5 challenge provided widely used benchmark networks with expression datasets (http://wiki.c2b2.columbia.edu/dream/index.php/D5c4) and gold standards. The E. coli dataset includes 334 TFs, 4511 target genes and 805 chips. In this study, we chose four subsets of different sizes from E. coli., i.e., including 92, 202, 1505 and 4511 genes. If the size of network is less than 4511, we simply selected genes at random. In this study, the gold-standard networks for all subsets are extracted from the overall gold-standard networks presented by DREAM5. We also selected one published dataset that contains more than 10 thousand genes [15].

### 3.2 Performance Evaluation 

#### 3.2.1 Result evaluation by different indexes

To evaluate the performance of LSGPA, four well-known indexes, namely true positive rate (TPR), false positive rate (FPR), positive predictive value (PPV) and accuracy (ACC) which are presented in S2 Text were calculated. To consider the overall performance of LSGPA, we also plot the receiver operating characteristic (ROC) curves. The initial networks with sizes from 92 to 4511 are depicted in S3–S6 Figs, respectively. The networks inferred by LSGPA are listed in S7–S10 Figs, and the details for those networks are shown in S2–S5 Tables, Moreover, the results for the different sizes are listed and compared in Tables 2–5, respectively.

**Table 2:** Comparison of four indexes on network with size 92.

Methods	TPR	FPR	PPV	ACC
LSGPA	**0.7545**	**0.0443**	0.1316	**0.9553**
NARROMI	0.7000	0.0500	**0.1360**	0.9220
PCA-CMI(0-order)	0.7222	0.1105	0.0095	0.8894
PCA-CMI(1-order)	0.7182	0.3777	0.0028	0.6225
PCA-CMI(2-order)	0.7182	0.3777	0.0028	0.6225

Remark: In all tables, the best results for the relative items are noted in bold.

**Table 3:** Comparison of four indexes on network with size 202.

Method	TPR	FPR	PPV	ACC
LSGPA	**0.6000**	**7.7257e-04**	**0.0882**	**0.9992**
NARROMI	0.1837	0.0123	0.0176	0.9867
PCA-CMI(0-order)	0.1224	0.3426	4.2502e-04	0.6531
PCA-CMI(1-order)	0.2653	0.4003	0.0008	0.7993
PCA-CMI(2-order)	0.2653	0.4003	0.0008	0.7993

Remark: In all tables, the best results for the relative items are noted in bold.

**Table 4:** Comparison of four indexes on network with size 1505.

Method	TPR	FPR	PPV	ACC
LSGPA	0.4179	**0.0016**	**0.0573**	0.9983
NARROMI	0.1770	3.1266e-4	0.0139	**0.9994**
PCA-CMI(0-order)	0.4240	0.3633	2.4162e-4	0.6366
PCA-CMI(1-order)	**0.4664**	0.4056	0.0003	0.5943
PCA-CMI(2-order)	non	non	non	non

**Table 5:** Comparison of four indexes on network with size 4511.

Method	TPR	FPR	PPV	ACC
LSGPA	**0.4840**	**3.6150e-04**	**0.0863**	**0.9996**
NARROMI	0.2780	0.0007	0.0650	0.9987
PCA-CMI(0-order)	0.3819	0.3238	0.0020	0.6762
PCA-CMI(1-order)	non	non	non	non
PCA-CMI(2-order)	non	non	non	non

From Table 2, we can observe that LSGPA performed almost the best among prominent methods including NARROMI [14] and the CMI-based path consistency algorithm [12]. For PCA-CMI, we calculated three cases in the zero-order, first-order and second-order. Tables 3–5 also show that our method significantly outperforms these popular methods in terms of false positives and accuracy. In particular, with increasing network size, the first-order and second-order PCA-CMI almost cannot work. Although zero-order PCA-CMI can obtain the results, the accuracy is quite low. These results demonstrated that LSGPA is suitable for reconstructing large-scale networks.

**Fig 3:**
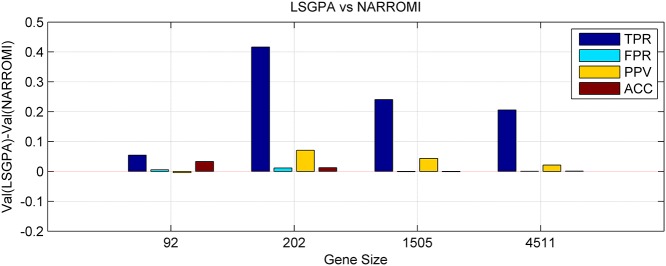
Difference between LSGPA and the two methods LSGPA and NARROMI in four indexes. Val() in vertical axis represents one of the four indexes for different methods.

**Fig 4:**
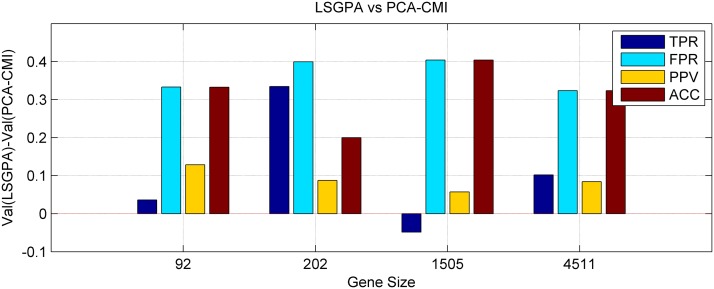
Difference of two methods LSGPA and PCA-CMI in four indexes.

Figs. 3 and 4 depicted the difference of the LSGPA with other two methods NARROMI and PCA-CMI based on the four indexes, i.e., using the indicator value for the proposed LSGPA minus the value for other methods. We can clearly see that the vast majority of the comparison values are over the zero line, which means that the performance of our algorithm is much better than the other methods, especially in larger sets.

Fig. 4 is more obvious: almost all the indexes from size 92 to 4511 are above zero except for TPR at size 1505, that is to say, LSGPA is widely superior.

**Fig 5:**
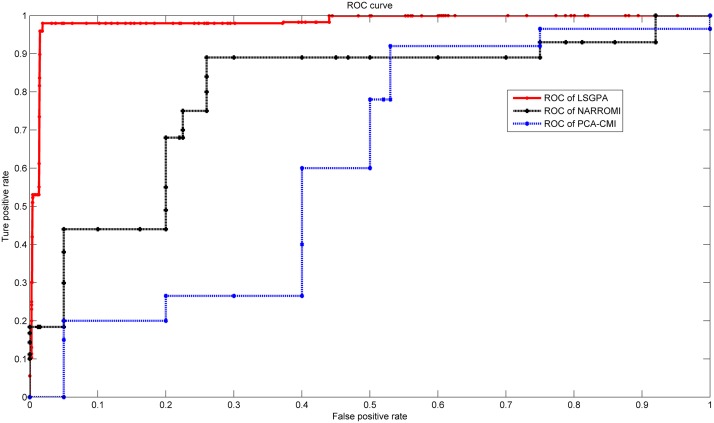
ROC curves of LSGPA on DREAM5 in size 202.

**Fig 6:**
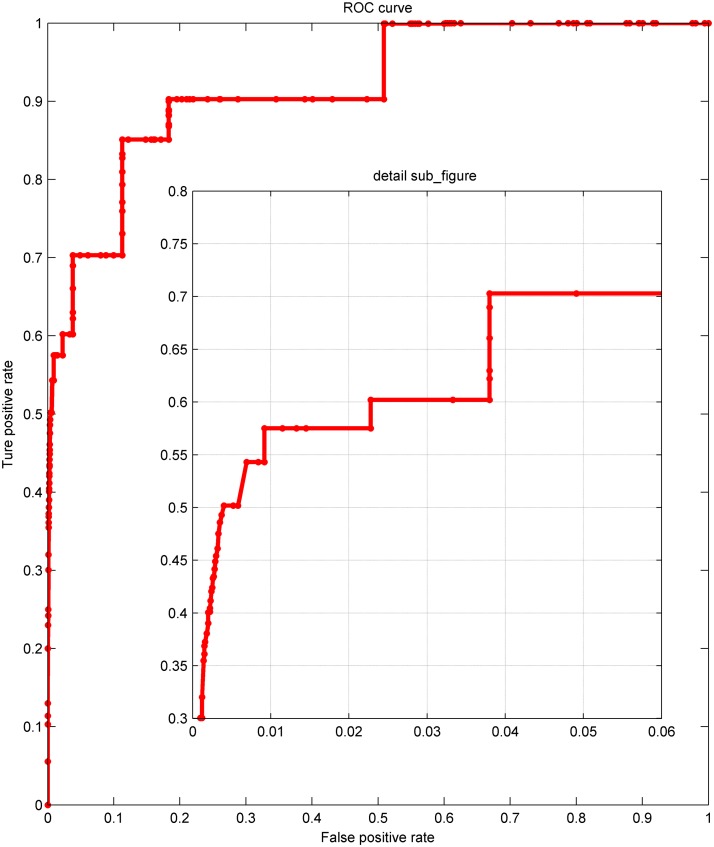
ROC curves of LSGPA on DREAM5 in size 1505. The subfigure shows the details.

Figs. 5 and 6 displayed the overall performance of LSGPA with size 202 and 1505. these ROC curves indicate that LSGPA has reached a very high level. Because of the unacceptable runtime for calculating the ROC of NARROMI and PCA-CMI in size 1505, we just depicted the curve of LSGPA in Fig. 6.

#### 3.2.2 The stability of the performance

To test the stability of the performance of LSGPA, we selected different truncated threshold values *θ* of the identificated parameters in line (**) in the pseudocode procedure in section 2.4.3

**Fig 7:**
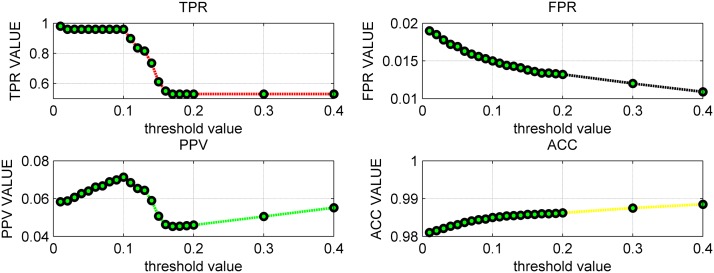
The calculated indexes for different parameter thresholds *θ* with size 202.

In Fig. 7 and S6 Table, the parameter threshold *θ* changed from 0 to 0.4 with 0.01 as the step length. Focusing on the TPR, it decreased with the increase of the threshold but stopped decreasing from 0.18 at 0.5306. FPR, PPV and ACC are all in the ideal state, for example, ACC varied from 0.9810 to 0.9885, and PPV changed between 0.0583 and 0.0552. Table 6 showed that the variances of the four indexes are all small, indicating the performance of LSGPA is not greatly influenced by the threshold value. For the size 1505, Fig. 8 (the detailed data are listed in S7 Table) and Table 7 demonstrated that all four indexes also maintained a stable level with some small fluctuations of the threshold value *θ*.

**Table 6:** Statistical analysis of four indexes on network with size 202.

Indexes	TPR	FPR	PPV	ACC
Maximum	0.9796	0.0190	0.0714	0.9885
Minimum	0.5306	0.0109	0.0453	0.9810
Variance	0.0382	4.2672e-06	8.1676e-05	3.4771e-06

**Fig 8:**
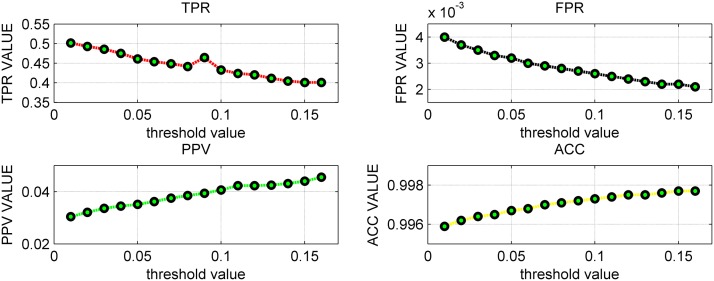
The calculated indexes for different thresholds *θ* with size 1505.

**Table 7:** Statistical analysis four indexes on network with size 1505.

Indexes	TPR	FPR	PPV	ACC
Maximum	0.5018	0.0040	0.0455	0.9977
Minimum	0.4011	0.0021	0.0304	0.9959

#### 3.2.3 Comparisons of runtime and speed up

To measure the efficiency of LSGPA, the runtime and speed up of the algorithm were recorded and compared. In this section, we first compared the time consumption of LSGPA and two famous methods NARROMI and PCA-CMI by running them on the same computer environment. The method for calculating the runtime is shown in S4 Text.

Furthermore, we computed the speed up of LSGPA using the following formula, i.e., the runtime of another method over the runtime of the LGSPA in the same computing environment.

Speed up=Runtime(Another Method)/Runtime(LSGPA) (11)

**Fig 9:**
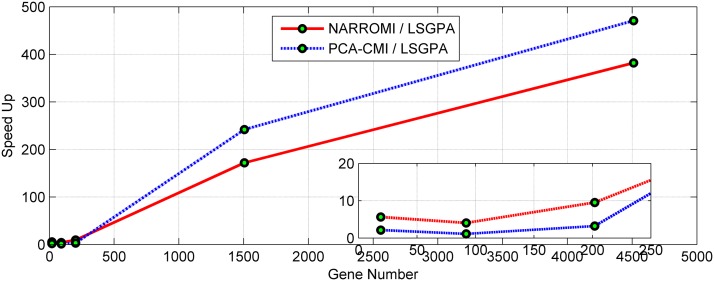
Speed up of the LSGPA against NARROMI and PCA-CIM for different gene numbers from E.coli.

This definition means that larger values correspond to better performance. Compared with NARROMI and PCA-CIM, Fig. 9 shows that as the network size increases, the speed-up of the LSGPA increases exponentially. These results indicate that the proposed parallel algorithm achieved a promising speed-up and thus can be used to handle large-scale data sets effectively.

**Fig 10:**
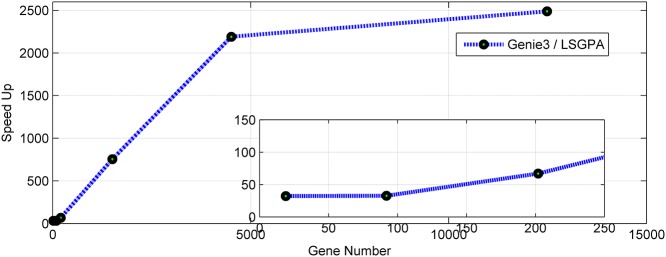
Speed up of the LSGPA against Genie3 for different gene numbers from datasets in [13].

To further prove its effectiveness, the LSGPA was also applied to construct GRNs from gene expression datasets in [15] with more than 10 thousand genes. In this dataset, there are 12488 target genes and 939 transfer factor genes, and 245 samples for each gene can be found in the expression dataset. Fig. 10 depicts the speedup of the LSGPA against Genie3 for different gene numbers in these datasets. The results further demonstrated the effectiveness of the LSGPA.

Table 8 lists the runtimes of different methods with different sizes. From size 92 to size 4511, LSGPA took less than 5 seconds, while NARROMI took over 1500 seconds, and PCA-CMI zero-order took almost 2000 seconds and also led to low accuracy. Note especially that in PCA-CMI, time cannot be counted for more than zero-order when dealing with sizes above 1505. These results indicate that the proposed algorithm is capable of handling large-scale data sets effectively.

**Table 8:** Runtimes for different sets with different sizes (seconds).

Methods\Sizes	92	202	1505	4511
LSGPA	**1.382400**	**1.654020**	**2.354940**	**4.183680**
NAROMI	5.606153	15.781613	404.341643	1597.859435
PCA-CMI(0-order)	1.530520	5.324213	569.249900	1969.614691
PCA-CMI(1-order)	56.062523	633.741694	2.6557e+005	non
PCA-CMI(2-order)	1943.272243	4.9291e+04	non	non

## Discussion and Conclusion

In this study, we proposed a novel asynchronous parallel framework (namely LSGPA) to improve the accuracy and lower the time complexity of large-scale GRN inference, by combining splitting technology and ordinary differential equation (ODE)-based optimization.

Our study makes three main contributions. First, we split the whole large-scale GRNs into many small-scale modular subnetworks by using the sparsity and modularity of large-scale GRNs. Second, combining ODE-based optimization of all subnetworks in parallel and their asynchronous communications, the connected subnetworks from all modules/Slaves are sent to the Master and the large-scale networks can be easily obtained. Third, we used different performance indexes, i.e., the well-known accuracy measures, stability measure, runtime and speedup to test the performance of the proposed approach in comparison with several popular algorithms on the same high-performance computing environments. The numerical results showed that the proposed LSGPA can be used effectively to infer large-scale GRNs with high precision and the computational time can be largely reduced.

Although the proposed LSGPA was mainly used for the reconstruction of GRNs, it can also be extended to infer the undirected networks. However, because of the complexity of connections and communications between different modules/ undirected subnetworks, we need to use the optimization algorithm to search for optimal connections between different Slaves [7, 26]. The detailed description is presented in S5 Text and the results are depicted in S11 Fig. The four indexes are compared in S8 Table. For the further work, we will make the dynamical analysis based on the constructed networks [27, 28].

In summary, we established a paradigm for inferring large-scale GRNs from large-scale high-throughput data by combining a splitting technique and optimization-based asynchronous parallel communications. The novel framework of the asynchronous parallel algorithm can be applied to solve related large-scale problems presented by large-scale omics data.

## Supporting Information

S1 Fig Example on undirected network. (TIF)

S2 Fig The selection of the MI threshold value *λ* on the structure of networks. (TIF)

S3 Fig Initial networks of Gene size 92. (TIF)

S4 Fig Initial networks of Gene size 202. (TIF)

S5 Fig Initial networks of Gene size 1505. (TIF)

S6 Fig Initial networks of Gene size 4511. (TIF)

S7 Fig Network with 92 genes inferred by the LSGPA (without isolated nodes). (TIF)

S8 Fig Network with 202 genes inferred by the LSGPA (without isolated nodes). (TIF)

S9 Fig Network with1505 genes inferred by the LSGPA (without isolated nodes). (TIF)

S10 Fig Network with 4511 genes inferred by the LSGPA (without isolated nodes). (TIF)

S11 Fig The undirected Network with 19 genes inferred by the LSGPA. (TIF)

S1 Table The selection of the MI threshold value *λ* on the structure of networks. (PDF)

S2 Table Details for the network with size 92. (PDF)

S3 Table Details for the network with size 202. (PDF)

S4 Table Details for the network with size 1505. (PDF)

S5 Table Details for the network with size 4511. (PDF)

S6 Table The effects of the threshold value *θ* of parameters in networks on the four indexes in size 202. (PDF)

S7 Table The effects of the parameter threshold value *θ* on the four indexes in size 1505. (PDF)

S8 Table Comparison of different indexes on network with size 19. (PDF)

S1 Text Install of MPI. (PDF)

S2 Text The calculation of different indexes. (PDF)

S3 Text Method to deal with the limitation of computer nodes. (PDF)

S4 Text The Calculation of runtime. (PDF)

S5 Text Application on undirected networks. (PDF)
