# Supplementary figures and images for: A New Asynchronous Parallel Algorithm for Inferring Large-Scale Gene Regulatory Networks
Source: PLoS One. 2015 Mar 25;10(3):e0119294. doi: 10.1371/journal.pone.0119294 (PMC4373852; doi:10.1371/journal.pone.0119294)

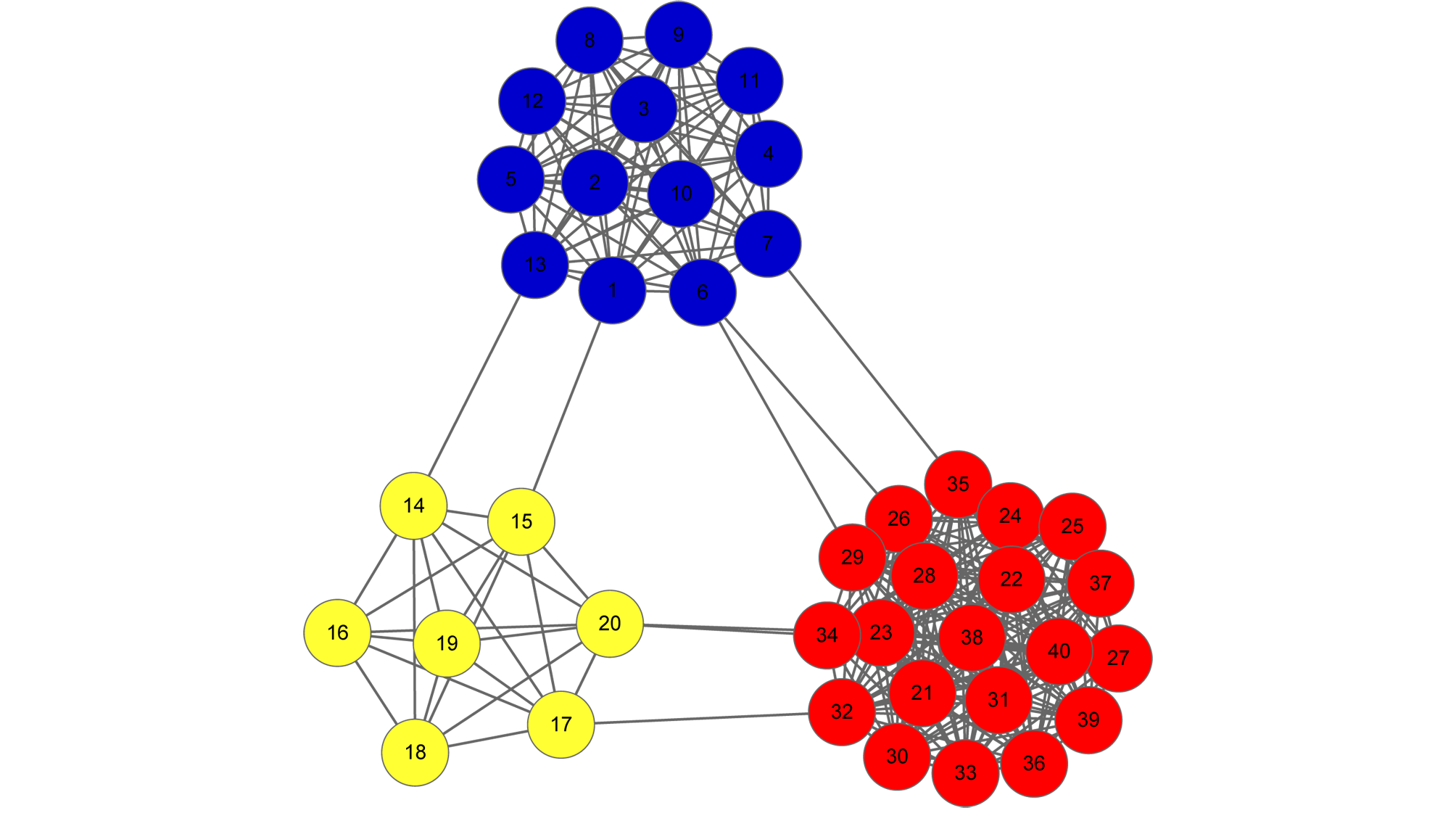

Supplement: S1 Fig — (TIF) [file pone.0119294.s001.tif]

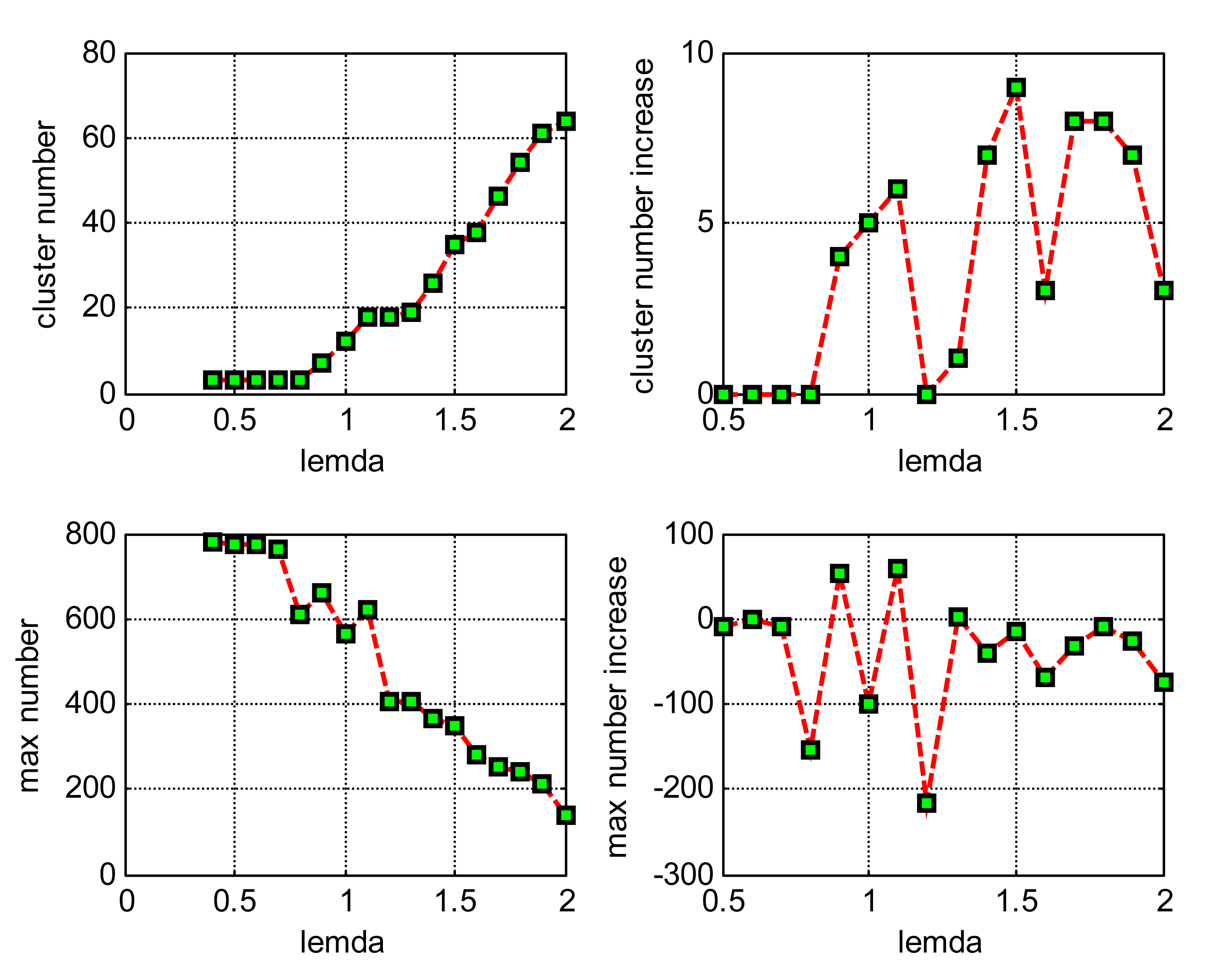

Supplement: S2 Fig — (TIF) [file pone.0119294.s002.tif]

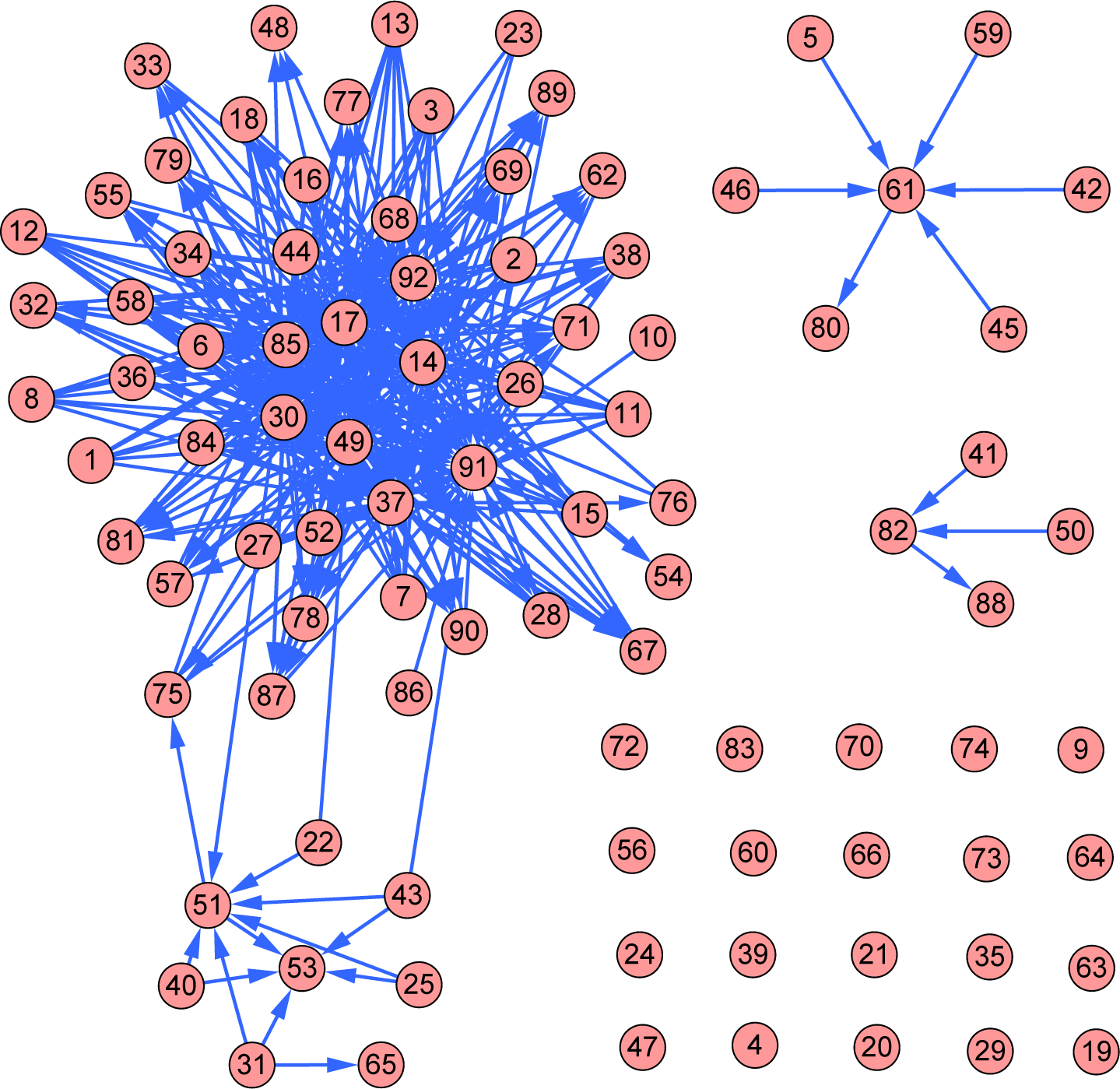

Supplement: S3 Fig — (TIF) [file pone.0119294.s003.tif]

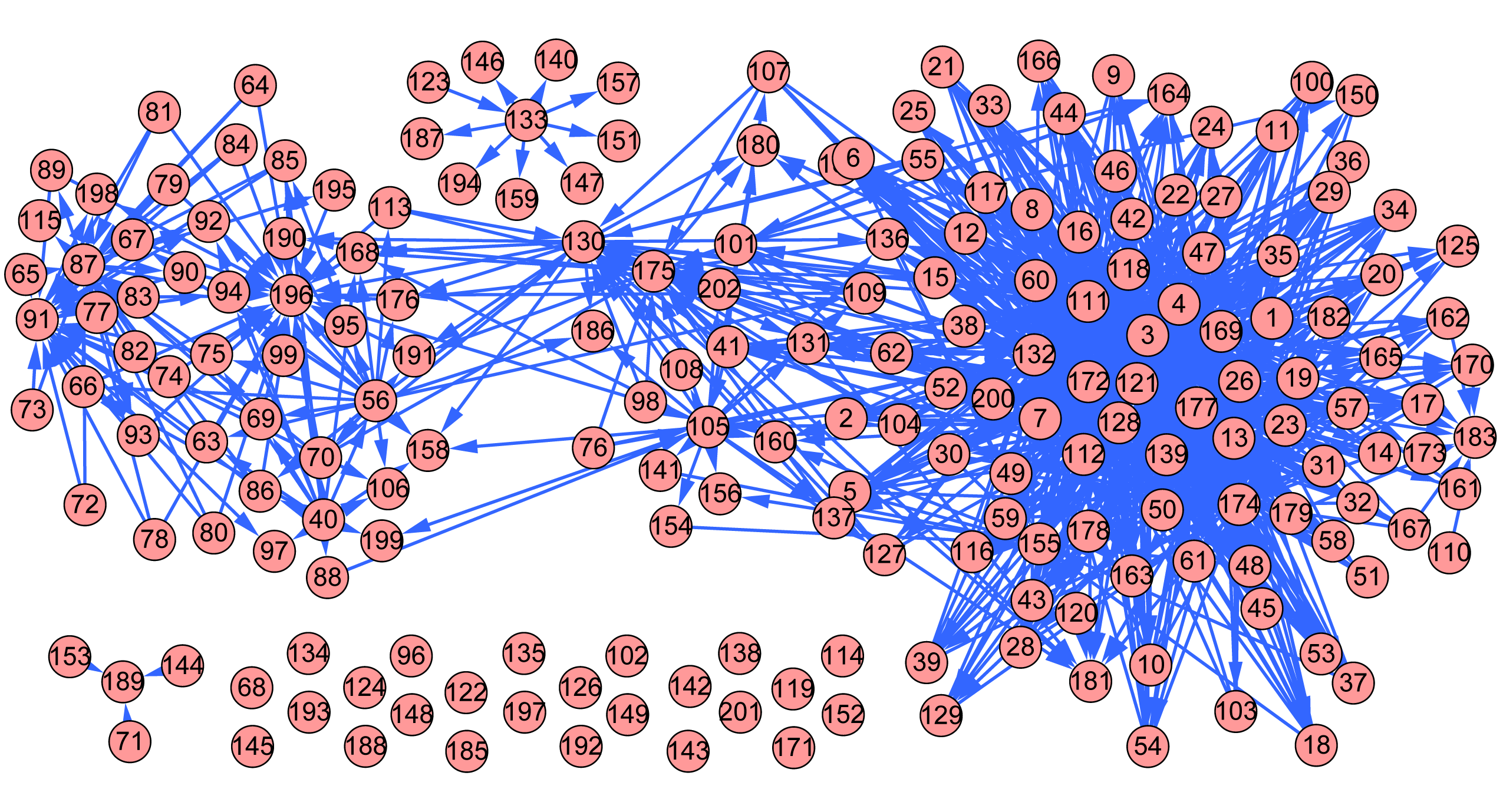

Supplement: S4 Fig — (TIF) [file pone.0119294.s004.tif]

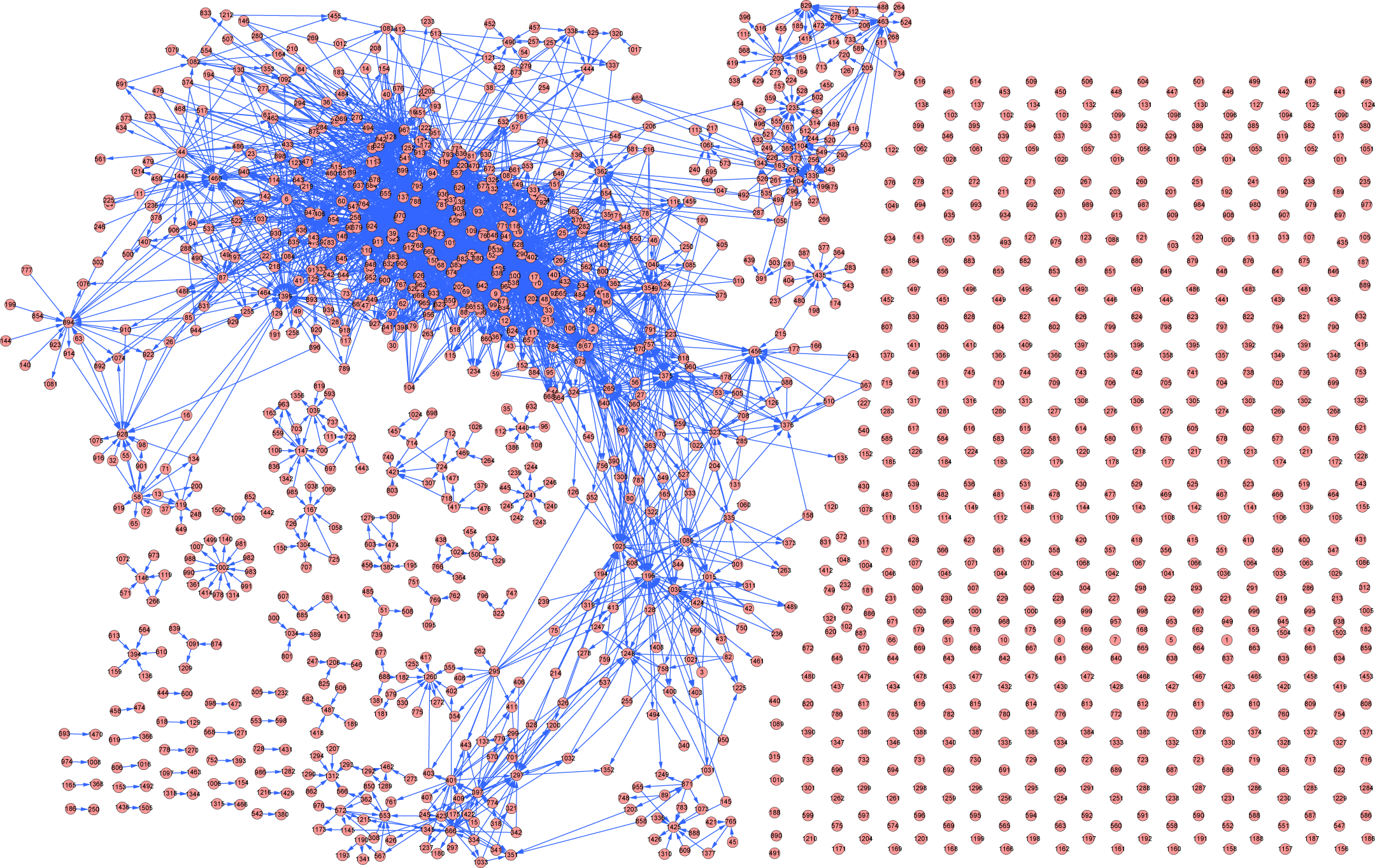

Supplement: S5 Fig — (TIF) [file pone.0119294.s005.tif]

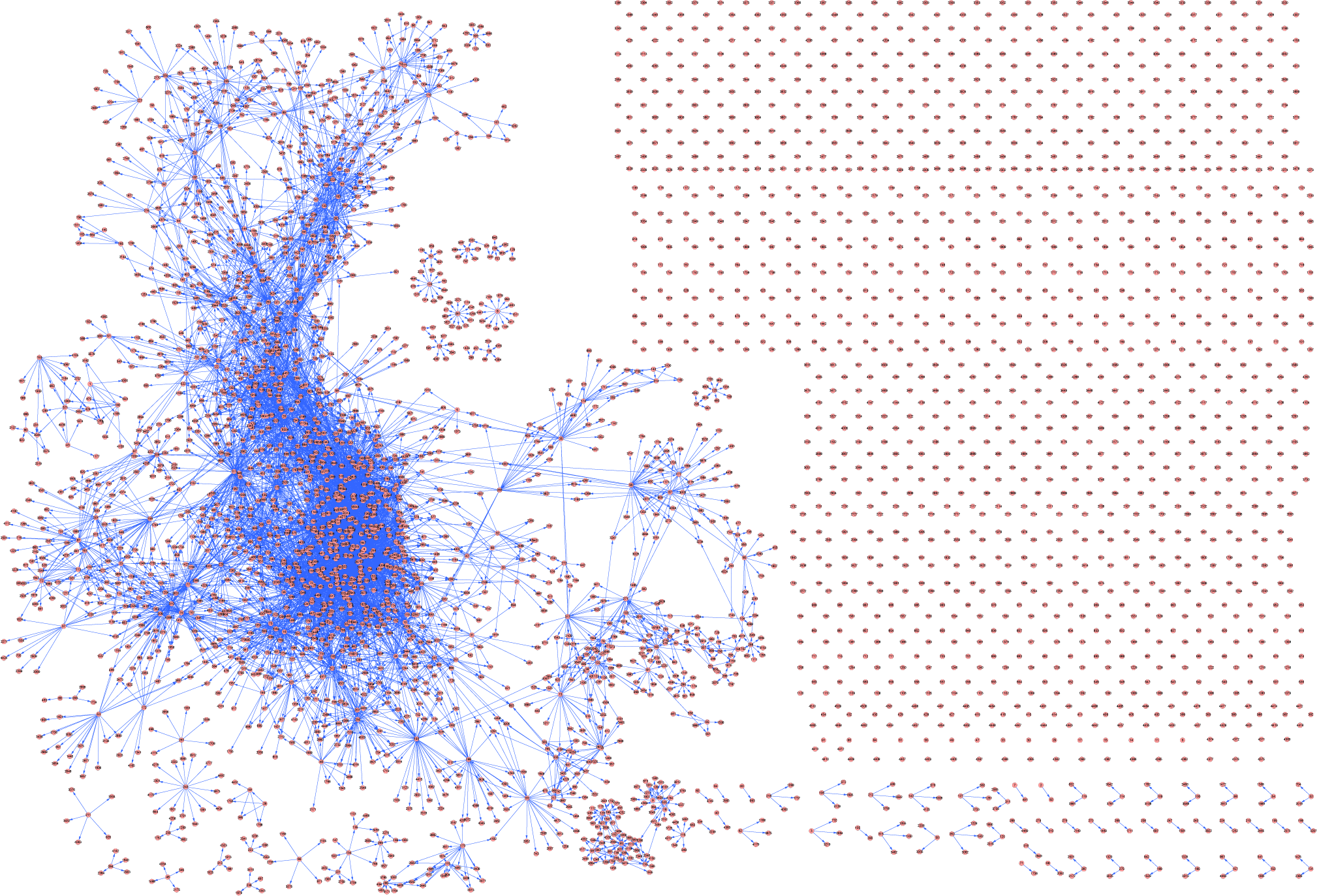

Supplement: S6 Fig — (TIF) [file pone.0119294.s006.tif]

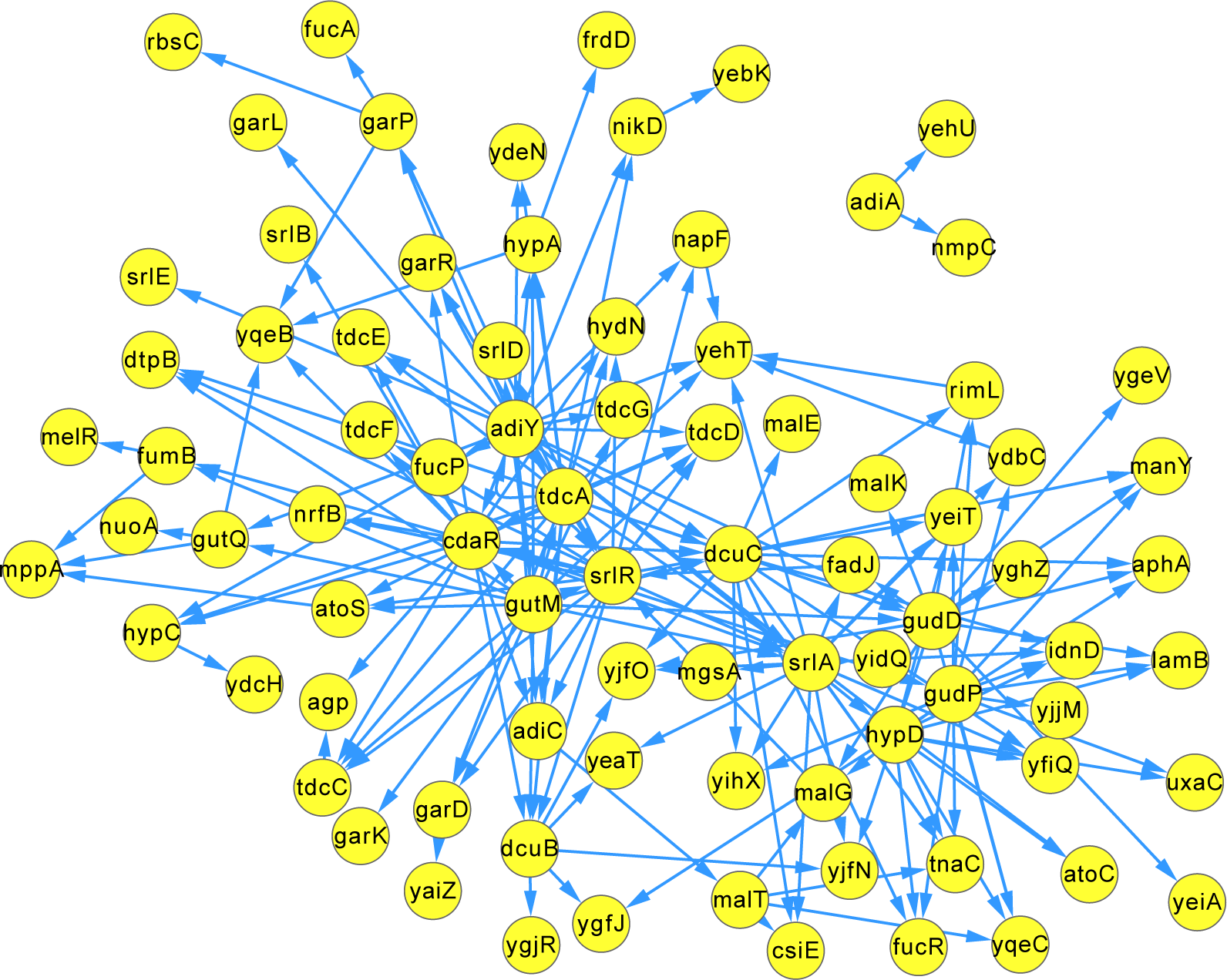

Supplement: S7 Fig — (TIF) [file pone.0119294.s007.tif]

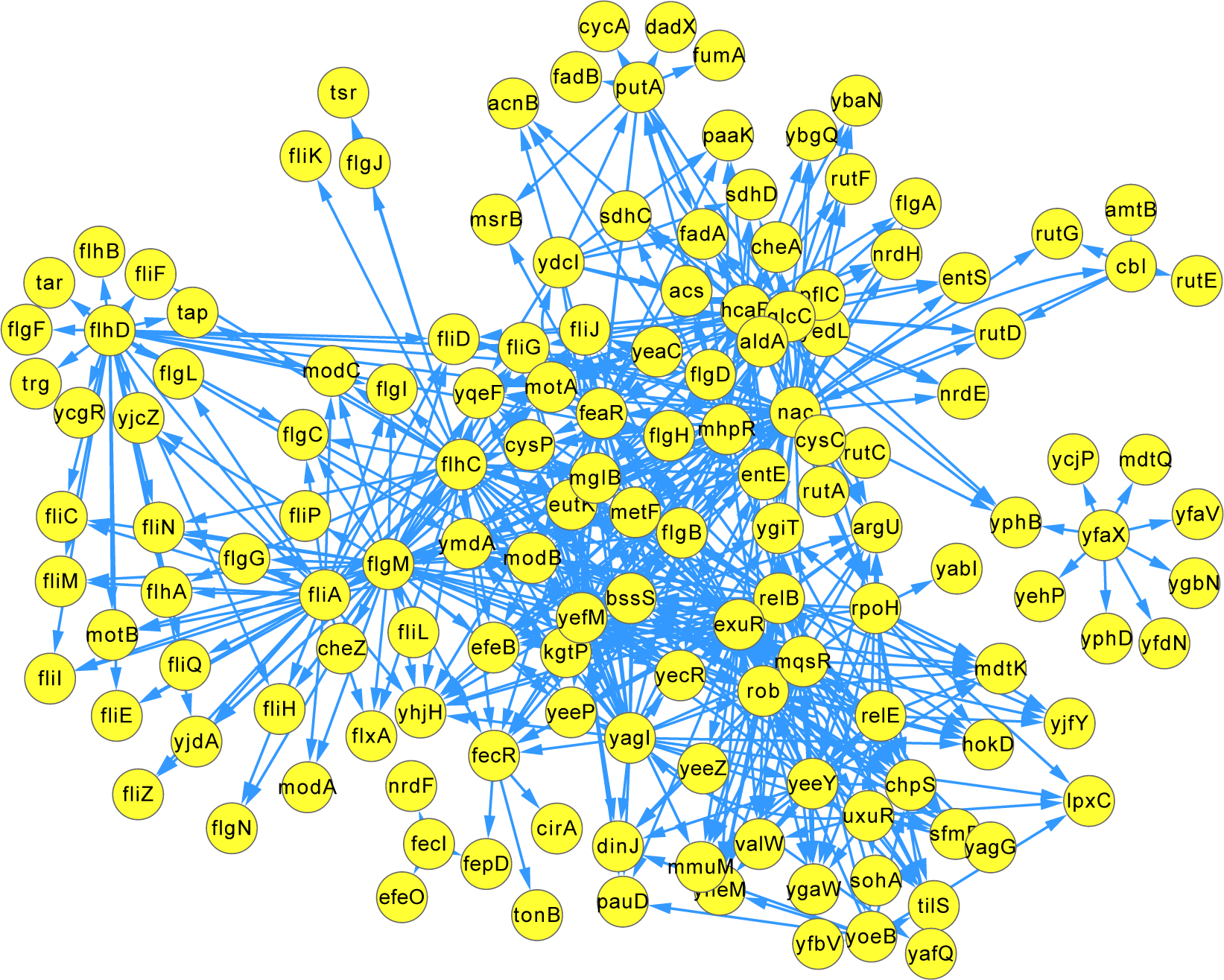

Supplement: S8 Fig — (TIF) [file pone.0119294.s008.tif]

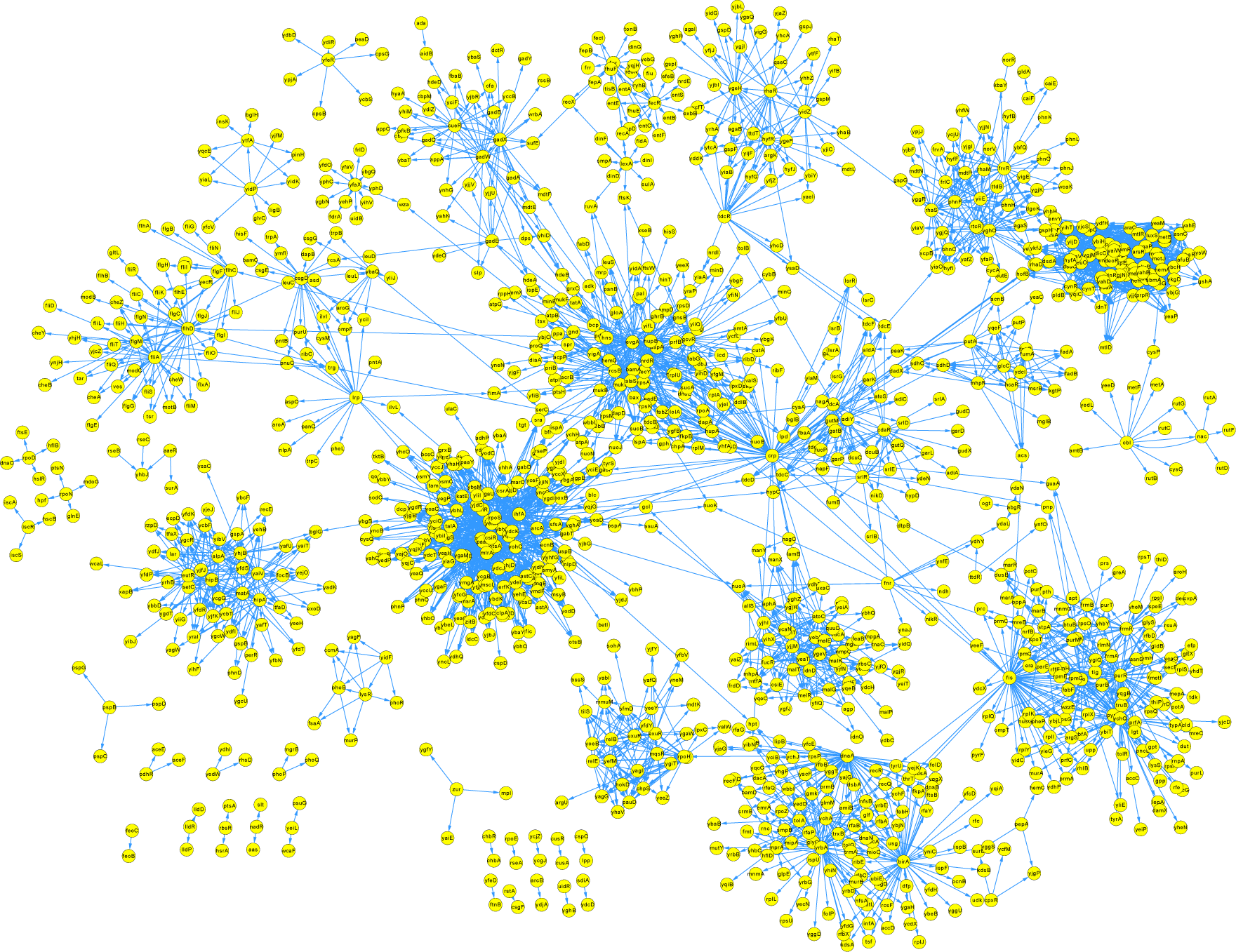

Supplement: S9 Fig — (TIF) [file pone.0119294.s009.tif]

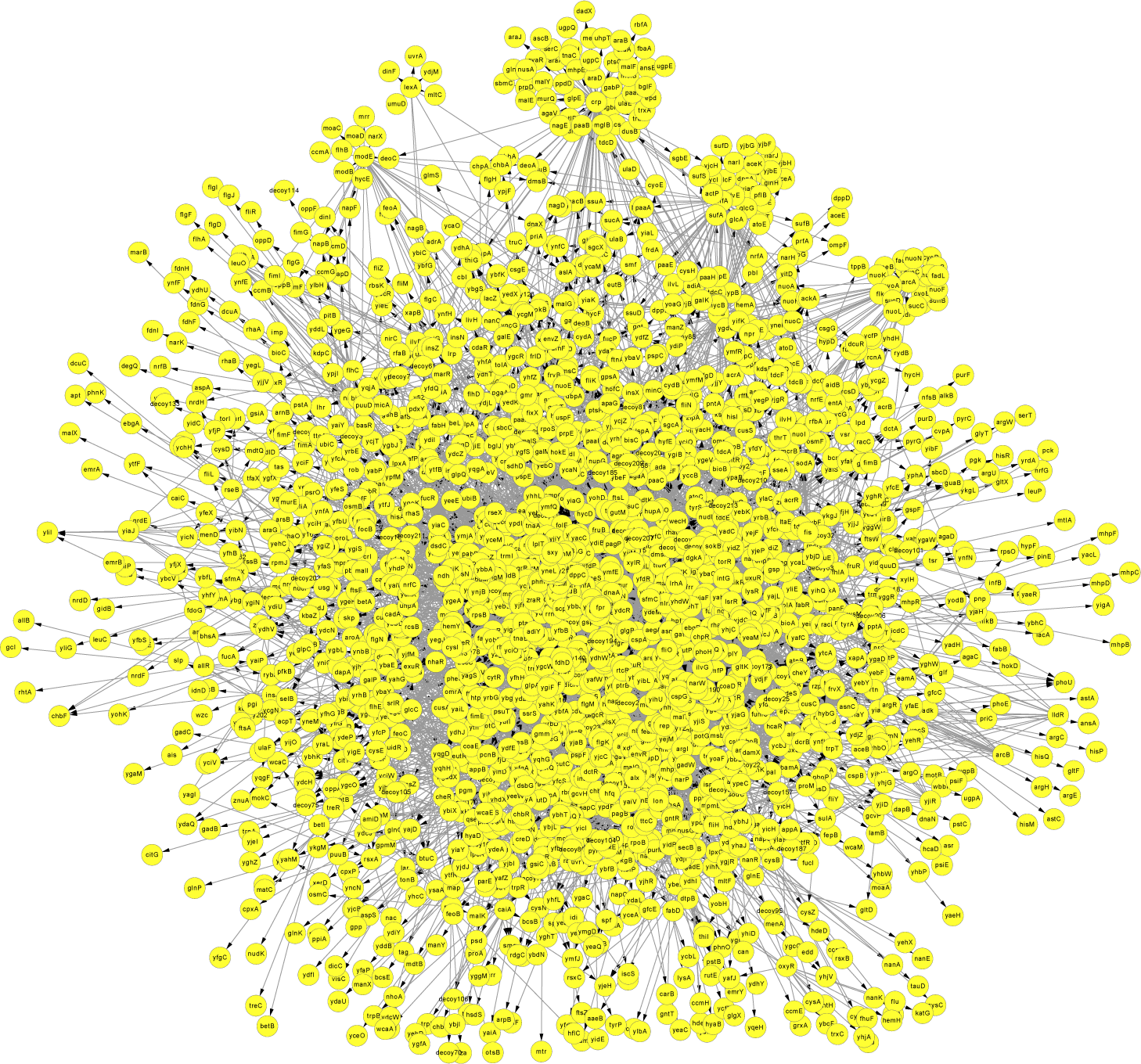

Supplement: S10 Fig — (TIF) [file pone.0119294.s010.tif]

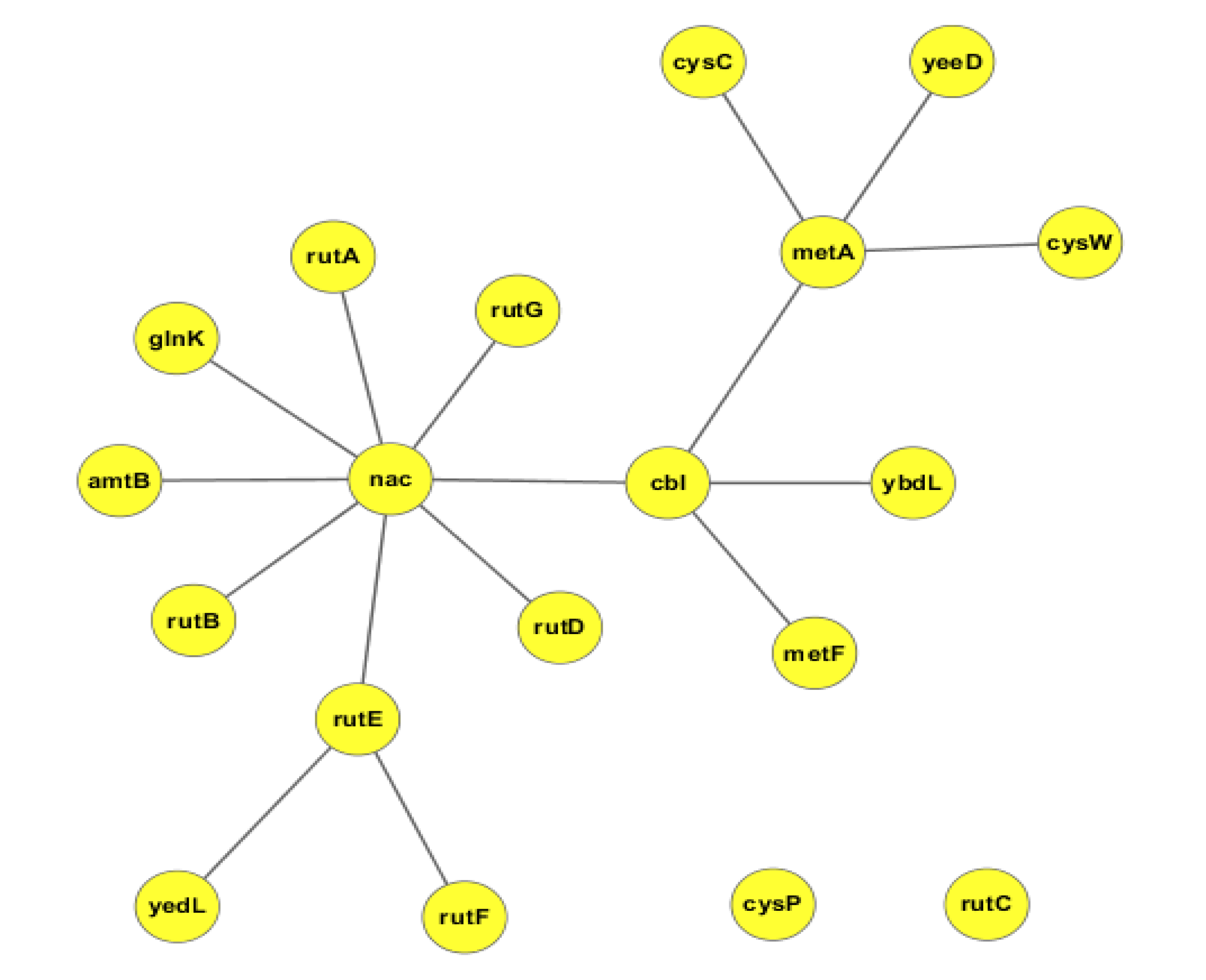

Supplement: S11 Fig — (TIF) [file pone.0119294.s011.tif]
