# Supplementary material for: A New Asynchronous Parallel Algorithm for Inferring Large-Scale Gene Regulatory Networks
Source: PLoS One. 2015 Mar 25;10(3):e0119294. doi: 10.1371/journal.pone.0119294 (PMC4373852; doi:10.1371/journal.pone.0119294)
Supplement: S1 Table — (PDF) [file pone.0119294.s012.pdf]

S1 Table. The selection of the MI threshold value λ on the structure of networks.

λ	clusters	Average	Max	Min	modularity	modularity	nodes
0.4	3	500.333	782	41	-0.201	0.166	1501
0.5	3	499.667	772	32	-0.178	0.184	1499
0.6	3	499.667	772	32	-0.178	0.184	1499
0.7	3	495.333	763	186	-0.084	0.224	1486
0.8	3	490	608	266	0.028	0.273	1470
0.9	7	206.857	661	2	0.006	0.272	1448
1.0	12	118.5	562	2	0.103	0.305	1422
1.1	18	76.889	621	2	0.074	0.298	1384
1.2	18	73.889	405	2	0.155	0.362	1330
1.3	19	67.421	407	2	0.242	0.383	1281
1.4	26	47.115	367	2	0.219	0.397	1225
1.5	35	33.257	351	2	0.244	0.416	1164
1.6	38	28.605	282	2	0.292	0.442	1087
1.7	46	21.761	250	2	0.331	0.467	1001
1.8	54	16.963	241	2	0.365	0.484	916
1.9	61	13.328	215	2	0.43	0.533	813
2.0	64	11.156	140	2	0.481	0.576	714
