# Supplementary material for: A New Asynchronous Parallel Algorithm for Inferring Large-Scale Gene Regulatory Networks
Source: PLoS One. 2015 Mar 25;10(3):e0119294. doi: 10.1371/journal.pone.0119294 (PMC4373852; doi:10.1371/journal.pone.0119294)
Supplement: S3 Table — (PDF) [file pone.0119294.s014.pdf]

**S3 Table. Details for the network with size 202**

| nodes | edges | isolated nodes |
| --- | --- | --- |
| 202 | 547 | 58 |
