# Supplementary material for: A New Asynchronous Parallel Algorithm for Inferring Large-Scale Gene Regulatory Networks
Source: PLoS One. 2015 Mar 25;10(3):e0119294. doi: 10.1371/journal.pone.0119294 (PMC4373852; doi:10.1371/journal.pone.0119294)
Supplement: S4 Table — (PDF) [file pone.0119294.s015.pdf]

S4 Table. Details for the network with size 1505

nodes	edges	isolated nodes
1505	3753	267
