# Supplementary material for: A New Asynchronous Parallel Algorithm for Inferring Large-Scale Gene Regulatory Networks
Source: PLoS One. 2015 Mar 25;10(3):e0119294. doi: 10.1371/journal.pone.0119294 (PMC4373852; doi:10.1371/journal.pone.0119294)
Supplement: S6 Table — (PDF) [file pone.0119294.s017.pdf]

S6 Table. The effects of the threshold value θ of parameters in networks on the four indexes in size 202

θ	TPR	FPR	PPV	ACC
0.01	0.9796	0.0190	0.0583	0.9810
0.02	0.9592	0.0185	0.0588	0.9815
0.03	0.9592	0.0178	0.0607	0.9821
0.04	0.9592	0.0172	0.0627	0.9827
0.05	0.9592	0.0169	0.0640	0.9831
0.06	0.9592	0.0163	0.0661	0.9837
0.07	0.9592	0.0159	0.0667	0.9841
0.08	0.9592	0.0156	0.0689	0.9844
0.09	0.9592	0.0153	0.0699	0.9846
0.10	0.9592	0.0150	0.0714	0.9850
0.11	0.8980	0.0147	0.0685	0.9852
0.12	0.8367	0.0144	0.0654	0.9854
0.13	0.8163	0.0143	0.0644	0.9855
0.14	0.7347	0.0141	0.0590	0.9856
0.15	0.6122	0.0138	0.0507	0.9858
0.16	0.5510	0.0136	0.0463	0.9858
0.17	0.5306	0.0134	0.0453	0.9860
0.18	0.5306	0.0134	0.0454	0.9860
0.19	0.5306	0.0133	0.0457	0.9861
0.20	0.5306	0.0132	0.0460	0.9862
0.30	0.5306	0.0120	0.0505	0.9875
0.40	0.5306	0.0109	0.0552	0.9885
