# Supplementary material for: A New Asynchronous Parallel Algorithm for Inferring Large-Scale Gene Regulatory Networks
Source: PLoS One. 2015 Mar 25;10(3):e0119294. doi: 10.1371/journal.pone.0119294 (PMC4373852; doi:10.1371/journal.pone.0119294)
Supplement: S8 Table — (PDF) [file pone.0119294.s019.pdf]

S8 Table. Comparison of different indexes on network with size 19

Method	TPR	FPR	PPV	ACC
LSGPA	**0.7222**	**0.0029**	0.4928	0.9134
NARROMI	0.7222	0.0262	**0.5909**	**0.9612**
PCA-CMI(0-order)	0.5000	0.4140	0.0596	0.5817
PCA-CMI(1-order)	0.5000	0.3469	0.0703	0.6454
PCA-CMI(2-order)	0.5000	0.3469	0.0703	0.6454

The best performer for the relative item is noted in bold.
