# Supplementary material for: A New Asynchronous Parallel Algorithm for Inferring Large-Scale Gene Regulatory Networks
Source: PLoS One. 2015 Mar 25;10(3):e0119294. doi: 10.1371/journal.pone.0119294 (PMC4373852; doi:10.1371/journal.pone.0119294)
Supplement: S1 Text — (PDF) [file pone.0119294.s020.pdf]

S1 Text. Install of MPI

The following section described the steps that used to build parallel environment in LINUX- based system. We need the following prerequisites in advance.
1) The tar file mpich.tar.gz (which can be obtained from the official website)
2) A C compiler (A Fortran compiler if needed)

Step 1. Create a directory for MPI and add it in the home directory.

$ mkdir MPI

Step 2. Unpack the tar file.

$ tar xfz mpich.tar.gz

The directory MPI will contain a sub-directory named mpich.tar.gz.

Step 3. Choose tehe installation directory

$ mkdir mpich-install

Step 4. Choose a building directory

$ mkdir mpich

Now the MPI directory will contain three sub-directories named mpich.tar.gz, mpich and mpich-install.

Step 5. Configure MPICH, specifying the installation directory and running the configure script in the source directory.

$ cd

$ cd MPI/mpich

$/home/you/MPI/mpich.tar.gz/configure -prefix=/home/you/MPI/mpich-install

Step 6. Build MPICH

$ make

Step 7. Install the MPICH commands.

$ make install

Step 8. Add the bin directory to your path which you will use.

$ export PATH=/home/you/MPI/mpich-install/bin:$PATH

Based on the above 8 steps, the MPI will be successfully installed.
