# Supplementary material for: A New Asynchronous Parallel Algorithm for Inferring Large-Scale Gene Regulatory Networks
Source: PLoS One. 2015 Mar 25;10(3):e0119294. doi: 10.1371/journal.pone.0119294 (PMC4373852; doi:10.1371/journal.pone.0119294)
Supplement: S2 Text — (PDF) [file pone.0119294.s021.pdf]

S2 Text. The calculation of different indexes

The most widely used statistical measures are true positive (TP), false positive (FP), true negative (TN) and false negative (FN) values. Also, four famous measures true positive rate (TPR), false positive rate (FPR), positive predictive value (PPV) and accuracy (ACC) are frequently used to assess the performance of a method for network reconstruction. They are defined as follows.

$$TPR = \frac{TP}{TP + FN}$$

$$FPR = \frac{FP}{FP + TN}$$

$$PPV = \frac{TP}{TP + FP}$$

$$ACC = \frac{TP}{TP + FP + TN + FN}$$

Furthermore, all of these general measures are global error measures because those statistical methods evaluate the network inference performance as a whole, represented by a scalar value.
