# Supplementary material for: A New Asynchronous Parallel Algorithm for Inferring Large-Scale Gene Regulatory Networks
Source: PLoS One. 2015 Mar 25;10(3):e0119294. doi: 10.1371/journal.pone.0119294 (PMC4373852; doi:10.1371/journal.pone.0119294)
Supplement: S3 Text — (PDF) [file pone.0119294.s022.pdf]

S3 Text. Method to deal with the limitation of computer nodes

This process provides a widespread way to use LSGPA on the other machine which may contain different computing nodes. *Max_N* is the maximum number of computing nodes.

```
Master process:
Data input
→ Calculate MI value of between all gene pairs and get the MI matrix
→ Clustering genes through MI matrix, M clusters.
→   MPI_Broadcast M to all Slaves
→   If M<=Max_N
do{
→ Extract every cluster data
    {
      MPI_Send data1 to Slave 1.
      MPI_Send data2 to Slave 2.
      …
      MPI_Send dataN to Slave M.
        }
→  MPI_Revc Net 1 to Slave 1.
   MPI_Revc Net 2 to Slave 2.
   …
   MPI_Revc Net M to Slave M.
}
→   If M>Max_N
do{
→ Extract every cluster data
     {
     MPI_Send data1 to Slave 1% Max_N
     MPI_Send data2 to Slave 2% Max_N
       …
     MPI_Send dataN to Slave N% Max_N
        }
→  MPI_Revc Net 1 to Slave 1% Max_N
   MPI_Revc Net 2 to Slave 2% Max_N
   …
   MPI_Revc Net N to Slave N% Max_N
  }
Note: % said modular arithmetic, which is the largest Slave number was 169, when the block number is greater than Max_N, such as cluster(Max_N+1) assigned Slave 1, (Max_N +1)% of Max_N = Slave and cluster (Max_N+2) assigned Slave 2, ( Max_N+2)% of Max_N = Slave so cycle.
→ Combine Net1~N and finally we obtain Net
→ Out put Net
Slave process No.1:
→ MPI_Revc N from Master process.
→ If N<= Max_N
do
{
→ MPI_Revc data1 from Master process.
→   MPI_Revc data from other slave processes.(the data which other clusters act on Slave NO.1)
MPI_Send data to all Slaves(the data which Slave NO.1 act on other clusters)
→ Get Net 1 result from optimization method
→ MPI_Send Net 1 to Master Process
}
→ If N> Max_N
do{
```

→ MPI_Revc data1 from Master process.<br>→ MPI_Revc data from other slave processes.(the data which other clusters act on cluster NO.1)<br>MPI_Send data to all Slaves (the data which Slave clusterNO.1 act on other clusters)<br>→ Get Net 1 result from optimization method<br>→ MPI_Send Net 1 to Master Process<br>→ MPI_Revc data(Max_N+1)from Master process.<br>→ MPI_Revc data from other slave processes.(the data which other clusters act on cluster NO.(Max_N+1))<br>MPI_Send data to all Slaves(the data which Slave cluster(Max_N+1)act on other clusters)<br>→ Get Net (Max_N+1)result from optimization method<br>→ MPI_Send Net (Max_N+1) to Master Process<br>}
Slave 2~ Max_N repeat the same operation as Slave 1.<br>Slave process No.2:<br>Slave process No.3:<br>…<br>Slave process No. Max_N
