# Supplementary material for: A New Asynchronous Parallel Algorithm for Inferring Large-Scale Gene Regulatory Networks
Source: PLoS One. 2015 Mar 25;10(3):e0119294. doi: 10.1371/journal.pone.0119294 (PMC4373852; doi:10.1371/journal.pone.0119294)
Supplement: S4 Text — (PDF) [file pone.0119294.s023.pdf]

S4 Text. The Calculation of runtime.

The pseudocode of computing the runtime is as follows.

```
if (computing node==Master node)
  do
    double Master_start_time, Master_end_time;
    double t0;
    Master_start_time = MPI_Wtime()
          Master Process
    Master_end_time = MPI_Wtime()
        t0= Master_end_time- Master_start_time;
enddo

else if (computing node==Slave nodes)
do
    double Slave_start_time, Slave_end_time;
    double T[];
    for i=1:M
        Slave_start_time = MPI_Wtime()
          Slave i Process
  end for
        Slave_end_time = MPI_Wtime()
        T[i]= Slave_end_time- Slave_start_time;
enddo
```

$$T = t0 + \max_{i \in \{1,2 \cdots M\}} \{T[i]\}$$
