# Supplementary material for: A New Asynchronous Parallel Algorithm for Inferring Large-Scale Gene Regulatory Networks
Source: PLoS One. 2015 Mar 25;10(3):e0119294. doi: 10.1371/journal.pone.0119294 (PMC4373852; doi:10.1371/journal.pone.0119294)
Supplement: S5 Text — (PDF) [file pone.0119294.s024.pdf]

S5 Text. Application on undirected networks

LSGPA can also handle undirected networks. For the sake of simplicity, we have used a simple example (S1 Fig.) to show the extension of our algorithm. All steps are the same as for directed networks except the following important optimization process.

In S1 Fig, we supposed that there are three modules to be detected using the method in section 2.2, and the system can be described by the following equations:

$$\begin{cases} \frac{dX}{dt} = AX + b_1 y_x + c_1 z_x \\ \frac{dY}{dt} = BY + a_1 x_y + c_2 z_y \\ \frac{dZ}{dt} = CZ + a_2 x_z + b_2 y_z \end{cases} \tag{S1}$$

where X , Y and Z represent three modules of this network. In right term of each equation, the first term represents the adjacency matrix inside each module, the second and third terms represents the coefficients of links between different modules. Therefore, we need to find the links between different modules before the subnetworks are identified.

First, by differencing the left item of Eq. (S1), we obtain

$$\begin{cases} DX = AX + b_1 y_x + c_1 z_x \\ DY = BY + a_1 x_y + c_2 z_y \\ DZ = CZ + a_2 x_z + b_2 y_z \end{cases} \tag{S2}$$

For the Master/Slave MPI process, sets X , Y and Z will be sent to three relatively independent computer nodes, namely Slaves.

Second, because links between different modules for the undirected networks are different from the links for directed networks, we need to solve the following optimization problem to obtain the links between modules.

$$\min \begin{Bmatrix} \left\| \frac{X^{\exp} - X^{sim}}{X^{\exp}} \right\|_2 (A, b_1, c_1) + \left\| \frac{Y^{\exp} - Y^{sim}}{Y^{\exp}} \right\|_2 (B, b_1, c_2), \\ \left\| \frac{X^{\exp} - X^{sim}}{X^{\exp}} \right\|_2 (A, a_1, c_1) + \left\| \frac{Y^{\exp} - Y^{sim}}{Y^{\exp}} \right\|_2 (B, a_1, c_2) \end{Bmatrix} \tag{S3}$$

We applied this method to construct the undirected network on size 19, and the inferred network is shown in S11 Fig.
